# Enhanced D1 and D2 Inhibitions Induced by Low-Frequency Trains of Conditioning Stimuli: Differential Effects on H- and T-Reflexes and Possible Mechanisms

**DOI:** 10.1371/journal.pone.0121496

**Published:** 2015-03-25

**Authors:** Rinaldo André Mezzarane, Fernando Henrique Magalhães, Vitor Martins Chaud, Leonardo Abdala Elias, André Fabio Kohn

**Affiliations:** 1 Laboratory of Signal Processing and Motor Control, College of Physical Education, Universidade de Brasília—UnB, Brasília, Brazil; 2 Biomedical Engineering Laboratory, Escola Politécnica, PTC, Universidade de São Paulo, São Paulo, Brazil; 3 School of Arts, Sciences and Humanities—EACH, Universidade de São Paulo, São Paulo, Brazil; 4 Department of Electrical Engineering, Universidade Federal do Triângulo Mineiro—UFTM, Uberaba, Brazil; 5 Department of Biomedical Engineering, School of Electrical and Computer Engineering, University of Campinas—UNICAMP, Campinas, Brazil; Emory University, UNITED STATES

## Abstract

Mechanically evoked reflexes have been postulated to be less sensitive to presynaptic inhibition (PSI) than the H-reflex. This has implications on investigations of spinal cord neurophysiology that are based on the T-reflex. Preceding studies have shown an enhanced effect of PSI on the H-reflex when a train of ~10 conditioning stimuli at 1 Hz was applied to the nerve of the antagonist muscle. The main questions to be addressed in the present study are if indeed T-reflexes are less sensitive to PSI and whether (and to what extent and by what possible mechanisms) the effect of low frequency conditioning, found previously for the H-reflex, can be reproduced on T-reflexes from the soleus muscle. We explored two different conditioning-to-test (C-T) intervals: 15 and 100 ms (corresponding to D1 and D2 inhibitions, respectively). Test stimuli consisted of either electrical pulses applied to the posterior tibial nerve to elicit H-reflexes or mechanical percussion to the Achilles tendon to elicit T-reflexes. The 1 Hz train of conditioning electrical stimuli delivered to the common peroneal nerve induced a stronger effect of PSI as compared to a single conditioning pulse, for both reflexes (T and H), regardless of C-T-intervals. Moreover, the conditioning train of pulses (with respect to a single conditioning pulse) was proportionally more effective for T-reflexes as compared to H-reflexes (irrespective of the C-T interval), which might be associated with the differential contingent of Ia afferents activated by mechanical and electrical test stimuli. A conceivable explanation for the enhanced PSI effect in response to a train of stimuli is the occurrence of homosynaptic depression at synapses on inhibitory interneurons interposed within the PSI pathway. The present results add to the discussion of the sensitivity of the stretch reflex pathway to PSI and its functional role.

## Introduction

The excitability of the stretch reflex pathway can be assessed by either a mechanical (tendon percussion) or an electrical (percutaneous current pulse) stimulus. A reflex response elicited by a tendon tap is usually referred to as a T-reflex (or phasic stretch reflex), while a reflex response obtained from an electrical stimulation to a mixed muscle nerve is known as an H-reflex [1]. The advantage of T-reflexes over H-reflexes is their routine use in clinical assessments of spinal cord integrity, since a basic mechanical percussion does not require any sophisticated apparatus [2]. Thus, it is generally easier (and cheaper) to deliver a mechanical percussion as compared to an electrical stimulation if a simple clinical hammer is used.

Additionally, the phasic stretch reflex is more natural than an H-reflex as it may occur in brisk movements or when a moving limb encounters an unpredictable rigid obstacle. On the other hand, the main advantage of the H-reflex is the reliable control of stimulus efficacy and its reproducibility (one action potential is fired per afferent fiber; [3]), allowing a fairly well controlled experiment in a variety of protocols. Investigations on the modulation of both reflexes have been conducted during the performance of different motor tasks [4–8].

The differences between stretch and H-reflexes and their responsiveness to a variety of conditioning protocols have already been addressed in great detail [3, 9–13]. The current view is that mechanically evoked reflexes (such as the T-reflex) are less sensitive to presynaptic inhibition (PSI) conditioning than is its electrical homologous (H-reflex) [13]. A hypothesis suggested in the literature for this phenomenon is a post-tetanic potentiation of the Ia terminals due to the Ia afferent burst of spikes with a consequent reduction of the level of PSI [13, 14].

PSI can be induced by different types of conditioning. For instance, in conditioning-to-test (C-T) paradigms, it is possible to increase the PSI level on the soleus (SO) Ia terminals by delivering an electrical pulse to the common peroneal nerve (CPN), known as the conditioning stimulus, followed by the test stimulus applied to the posterior tibial nerve (PTN) which elicits the conditioned reflex response. Mizuno et al. [15] identified two phases of inhibition in response to CPN stimulation, one termed D1 occurring at C-T intervals between 5 and 50 ms and a second one with C-T intervals between 70 and 200 ms called D2. While there is no agreement on which one corresponds to a pure PSI effect, both intervals have been used to study this inhibitory pathway [13, 16–22].

In their study on the sensitivity of H and T-reflexes to PSI, Morita et al. [13] focused on D1 inhibition. Curiously, a train of conditioning stimuli applied at 1 Hz was shown to strengthen the effect of D1 inhibition induced by CPN stimulation (applied 21 ms before the test stimulus) onto SO H-reflex [21]. Therefore, an interesting question to be posed is whether a similar train of conditioning stimuli would be able to induce a stronger inhibitory effect on the T-reflex as compared to a single conditioning stimulus. Based on previous studies [13, 14], one might expect the T-reflexes to be less responsive to the enhanced conditioning effect as compared to H-reflexes.

The effects described above for the C-T interval corresponding to D2 inhibition [15] have not yet been investigated. The C-T interval of ~100 ms has been used in a variety of protocols to induce PSI without the interference of postsynaptic effects [16, 18–20, 22]. The investigation of both D1 and D2 inhibitory responses has the potential to unravel differential mechanisms due to the different onset latency and time course for each inhibitory response.

From the considerations above, the present study aimed at: 1) investigating whether the effects of low frequency conditioning stimulation on H-reflexes found in previous work also occurs on T-reflexes; 2) describing possible differential effects on H- and T-reflexes; 3) examining if the sensitivity of both H- and T-reflexes to an “enhanced inhibition” is related to the C-T interval, i.e., D1 or D2 inhibition; 4) proposing possible underlying mechanisms for the PSI facilitation.

## Methods

### Participants

Nine healthy and right-footed volunteers (5 males and 4 females) aged 30 ± 7 yr (mean ± STD) participated in this study. All subjects gave written informed consent and all procedures were approved by the Human Ethics Committee of the Institute of Biomedical Sciences at the University of São Paulo. The experiments were conducted in accordance with the Declaration of Helsinki.

### Data acquisition and stimulation

The electromyograms (EMGs) were recorded using round-shaped surface electrodes (Ag-AgCl, 0.8 cm diameter, with an inter-electrode distance of 2 cm) applied in bipolar configuration over the SO and tibialis anterior (TA) muscles. The electrodes were positioned over the SO with the most proximal contact 4 cm beneath the inferior margin of the two heads of the gastrocnemii muscles. For TA, electrodes were positioned midway over the muscle belly. A ground electrode was placed over the tibia. The EMG signals were amplified and filtered (5 Hz to 2 kHz) by a MEB-2300K system (Nihon-Kohden, Japan) and then converted by an A/D board (National Instruments, Austin, TX.) at a 5 kHz sampling rate. Data were stored in hard disk for later off-line processing.

Direct motor responses (M-waves) and H-reflexes from the SO were obtained by electrical stimulation (rectangular pulses, 1 ms duration) applied to the posterior tibial nerve (PTN, test stimuli) delivered through surface electrodes (area = 2 cm^2^) positioned in the popliteal fossa [23]. For D1 and D2 studies, conditioning stimuli were applied to the common peroneal nerve (CPN) at the fibular head. Conditioning stimulus intensity was set at 1.1 times the motor threshold (1.1 x MT), with MT defined as the smallest stimulus intensity required to produce an observable M-wave of the TA.

**Fig 1:**
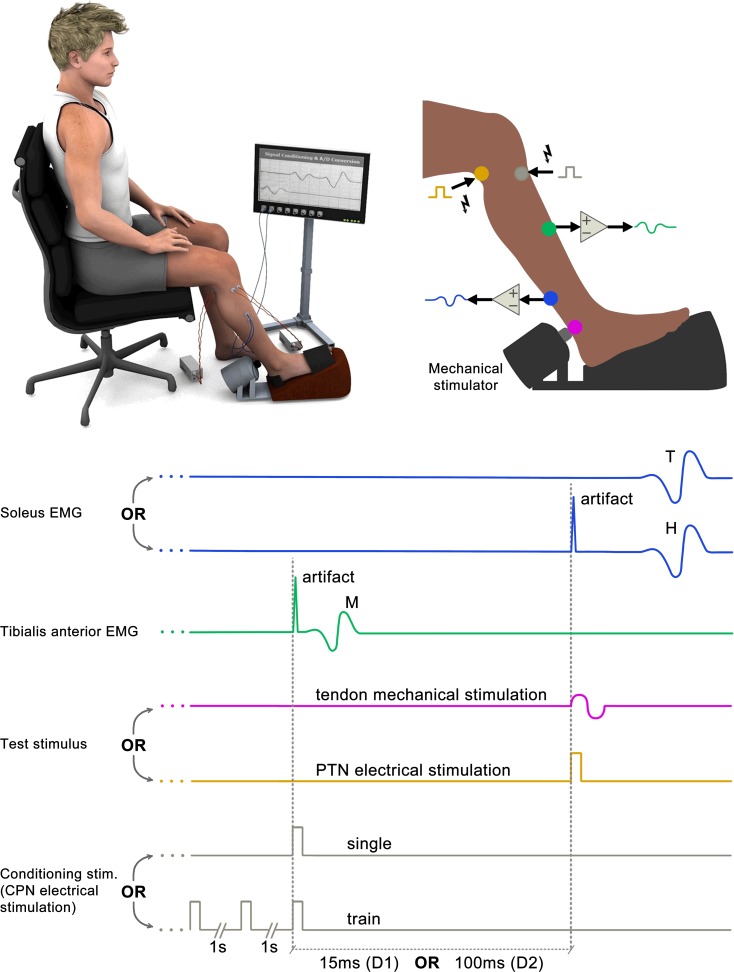
Schematic representation of the experimental setup and temporal organization of the experiment. Upper part: subject positioned during the experiment (left panel) showing the location of both the recording and the stimulation electrodes along with the shaker. In the right panel a detail of the stimulated leg and the points of both electrical (in gray and yellow) and mechanical (in magenta) stimuli delivery. The blue and green dots indicate the recording places for the SO and TA muscles, respectively. Lower part: schematic sweeps representing the EMG recordings and stimulus patterns whose colors are associated with the respective dots at the leg.

The mechanical test stimuli (to elicit the T-reflex) were applied to the Achilles tendon of the right leg by means of a LW-126-13 vibration system (Labworks, USA), consisting of a power amplifier and a shaker (cylindrical body, with diameter 10.5 cm and length 13.5 cm). The tip of the shaker (round-shaped plastic tip, 1 cm diameter) was pressed against the Achilles tendon in order to keep a steady pressure and a fixed position on the tendon [22, 24–26] (Fig. 1). A LabView system (National Instruments, Austin, TX) was utilized to generate one cycle of a sinusoid with a period of 10 ms [22, 24] which was delivered to the input of the shaker's power amplifier in order to obtain the desired parameters for the mechanical stimulation.

### General Procedures

Subjects were seated on a customized chair designed for stabilizing their right leg and feet. The hip, knee and ankle of both legs were maintained at ~90°. The shaker was on the floor in front of the chair (see the scheme in Fig. 1).

At the beginning of the experiments, five maximal motor waves (Mmax) were elicited at 0.5 Hz by delivering a supramaximal electrical stimulation to the PTN. Mmax and reflex amplitudes (i.e., T and H-reflexes) were estimated online from single unrectified EMG sweeps displayed on the screen of an oscilloscope (Hewlett Packard, Colorado Springs, CO). The intensities of the test stimuli used to evoke H and T-reflexes (electrical and mechanical stimuli, respectively) were adjusted so that the amplitude of the unconditioned response (i.e., control reflex) corresponded to ~20% of the amplitude of the Mmax (defined as the maximal peak-to-peak amplitude observed among the 5 Mmax).

It has been postulated that the test H-reflex in the 20–30% range of the maximal direct muscle response (Mmax) is more responsive to conditioning regardless of its nature [27]. We hypothesized that this holds true for T-reflexes as well. Thus, a device that allows the generation of T-reflexes with amplitudes approximately in the range given above was currently employed [8, 24–26] (see Data Acquisition and Stimulation).

To evaluate the effect of D1 inhibition, H-reflexes of the SO muscle were conditioned by either a single electrical pulse (1 ms duration) applied to the CPN (1.1 x MT) at C-T interval of 15 ms or a train of 7–10 pulses with the same duration/intensity delivered at 1 Hz, with the last pulse preceding the test stimulus by 15 ms. The number of pulses in the train varied pseudo-randomly within the range of 7 to 10 pulses in order to avoid, or at least minimize, a bias in the results due to a voluntary (or even an involuntary/subconscious) prediction of the time of the test pulse by the subject [21, 28]. For the same reason, the interval between test stimuli (applied to the PTN) pseudo-randomly ranged between 11 s and 13 s. The situations in which the reflexes were conditioned by a single pulse were termed “single”. The term “train” was employed when the reflexes were conditioned by a train of stimuli. The control H-reflex (without any conditioning), the H-reflex conditioned by a single pulse and the H-reflex conditioned by the train were evoked in a random fashion. Twenty reflex responses were obtained in each condition (i.e., train, single and control). The duration of the entire trial was ~13 min.

In another trial, a 100 ms C-T interval (instead of 15 ms) was used to evaluate the H-reflex under the effect of D2 inhibition. The same protocol described above was followed for T-reflexes, except that 3 to 6 ms were added to the C-T intervals (for both D1 and D2 trials) according to the subject’s height to compensate for the distance of the test stimulus applied to the triceps surae tendon [13].

The M-wave in the EMG of the TA muscle was constantly monitored to assure a steady intensity of the conditioning stimulation throughout the experiment. For some subjects, it was not possible to measure the M-wave amplitude of the TA EMG signals for the condition using C-T interval of 15 ms due to the interference from the PTN stimulus artifact (“crosstalk”). In those cases, the “crosstalked” stimulus artifact from the SO EMG in the control condition (with no conditioning and hence no M-wave in the TA EMG) was subtracted from the TA EMG in both PSI conditions (single and train) in order to reveal a clear and measurable M-wave. For these and all other cases, no differences in the amplitude of the TA M-wave were detected among conditions.

For each subject, the order of the trials (with T or H reflexes, D1 or D2 conditioning) was randomly presented and a resting period of ~5 min was allowed between trials. Each subject completed all trials in a single experimental session, which lasted ~3h.

Traces showing the temporal organization of the experiment are depicted in Fig. 1. The traces in blue color show schematic representations of both T- (upper traces) and H-reflexes (lower traces). TA M-wave is displayed in green and the mechanical and electrical test stimuli are shown in magenta and yellow, respectively. The bottom grey traces represent the conditioning stimuli (Fig. 1).

### Signal processing and data analysis

In order to evaluate the effect of conditioning (that induces either D1 or D2 inhibition) on both T and H-reflexes, the amount of inhibition (AOI) was measured for “single” and “train” conditions [21]. Therefore, conditioned H- and T-reflexes were expressed as the AOI, which consisted of the difference between control and conditioned reflex response over its control value, as follows:

$$AOI=\left(\frac{{\bar{H}}_{cont}-{\bar{H}}_{cond}}{{\bar{H}}_{cont}}\right)\times 100\%$$

in which the term ${\bar{H}}_{cont}$ refers to the mean control reflex amplitudes and ${\bar{H}}_{cond}$ is the mean reflex amplitudes conditioned by either a single or a train of electrical pulses to the CPN (n = 20 for each subject). Thus, the amount of inhibition for “single” (AOI_Singl) and “train” (AOI_Train) conditions were calculated for each subject. A three-way ANOVA with repeated measures [type of conditioning (“single” *vs* “train”), type of reflex (H- *vs* T-reflex) and type of inhibition (D1 *vs* D2)] was used to detect main effects and interactions among experimental conditions.

In order to evaluate the relative effect of the conditioning “train” with respect to the “single” conditioning, the ratio AOI_Train /AOI_ Singl (train to single ratio, TSR) was computed for both H- and T-reflexes and for both D1 and D2 inhibitions (a similar procedure has been adopted to compare healthy and hemiplegic patients by Roche et al. [21]). Here, we were interested in quantifying how much the reflex inhibition due to a conditioning train was higher than that due to a single stimulus conditioning.

The amplitude values of T-reflex in condition “single” obtained from two subjects were very similar to the respective control values. This resulted in T_AOI_Single values close to zero (very small effect of the single pulse) or even negative due to the intrinsic variability of the conditioned T-reflex itself. These negative and near-to-zero values found in two out of nine subjects strongly affected the ratio T_AOI_ Train/T_AOI_ Single, since values close to zero in the denominator yielded quite large ratio values. As negative values of this ratio are meaningless, data associated with those two subjects were removed from the analysis.

A two-way ANOVA with repeated measures was used to detect possible differences and interactions of TSR values between type of reflex (T *vs* H) and type of inhibition (D1 *vs* D2). The same statistical procedure (two-way ANOVA) was used to investigate possible differences in the amplitude of the control (unconditioned) H- and T- reflexes (expressed in %Mmax) measured during D1 and D2 trials.

All statistical analyses were performed using the statistical package SPSS 15.0 for Windows (SPSS, Inc., Chicago, IL), with significance level set at p < 0.05.

## Results

**Fig 2:**
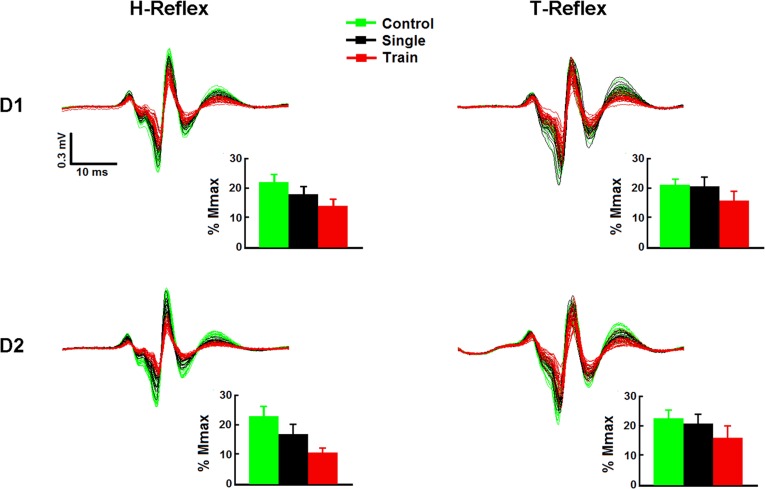
Raw data from one representative subject showing the effect of PSI on H- and T-reflexes at C-T intervals corresponding to D1 and D2. H- and T-reflexes are shown in the left and right panels, respectively. Each panel depicts 60 superimposed sweeps, including sweeps without conditioning (20 green traces, control reflexes), with single conditioning pulse (20 black traces, “single” responses) and conditioned by a train of pulses (20 traces in red, “train” responses) obtained from one subject. Bar graphs with the corresponding averaged reflex responses for this subject are also displayed. Care was taken to obtain control responses with similar amplitudes (20%Mmax) for both H- and T-reflexes (see the green bars). The effect of a single conditioning pulse on the T-reflex was noticeably weaker than that observed for the H-reflex (compare black and green bars), for both D1 (upper panels) and D2 (lower panels). Note the stronger effect of PSI induced by a train of stimuli as compared to a single conditioning pulse (compare red and black bars), for both reflexes regardless of D1 or D2 C-T intervals. The vertical traces indicate the standard error of the mean (SEM). The vertical and horizontal calibrations are identical for all sweeps.

Raw SO EMG showing H- and T-reflexes obtained from one representative subject under D1 and D2 trials (upper and lower panels, respectively), with respective mean bars drawn beneath, are presented in Fig. 2. One can notice an appreciable effect of a single pulse to CPN on the H-reflex (compare green and black sweeps and bars in the left column of Fig. 2). However, this inhibition is weaker for the T-reflex as compared to the H-reflex (right column of Fig. 2). On the other hand, the stronger effect of the train of conditioning stimuli compared to a single conditioning pulse is evident in this subject for both reflex responses and C-T intervals (compare the red and black sweeps and bars of Fig. 2).

**Fig 3:**
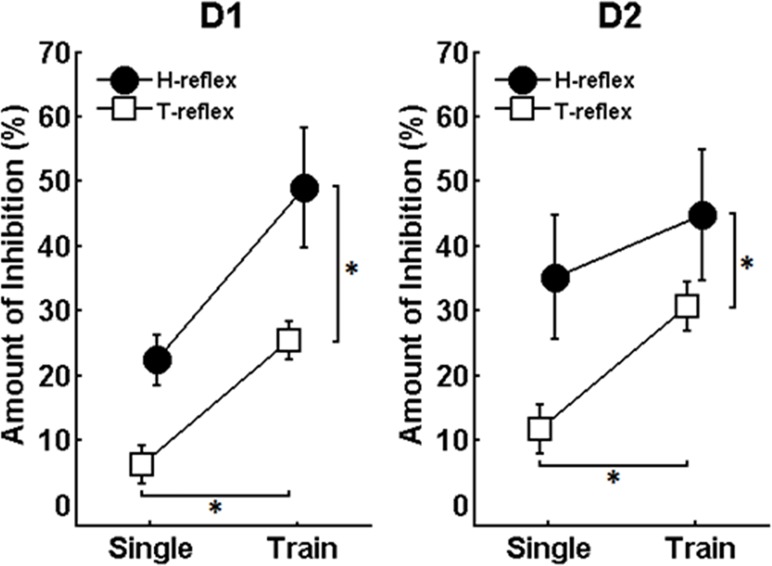
Amount of inhibition (AOI) for H- and T-reflexes in two different experimental conditions for both C-T intervals investigated (D1 and D2 inhibitions). The “train” condition always produced larger AOI as compared to the “single” condition irrespective of the probe (H- or T-reflex) or the C-T interval (p<0.001). H-reflexes were more prone to PSI, irrespective of the type of conditioning (“single” or “train”) or C-T interval (that induces D1 or D2 inhibition) (p = 0.007). Vertical traces are SEM. Asterisks indicate significant differences between conditions.

Fig. 3 shows the averaged values (n = 9) of AOI induced by “single” and “train” conditioning, for both T- and H- reflexes and for both C-T intervals (i.e. D1 and D2 inhibitions). The three-way ANOVA test performed on AOI measures revealed significant main effects for the factors “type of reflex” (F_(1,32)_ = 8.475, p = 0.007) and “type of conditioning” (F_(1,32)_ = 60.5, p < 0.001). No significant main effect was detected for the factor “type of inhibition (duration of C-T interval)” (F_(1,32)_ = 0.530, p = 0.472) and no significant interactions were found among conditions (p > 0.05). These results indicate that 1) H-reflexes were more prone to PSI inhibition than T-reflexes (regardless of the type of conditioning and C-T interval) and that 2) the “train” conditioning caused significantly larger inhibition than the “single” condition (regardless of the type of reflex and C-T interval).

**Fig 4:**
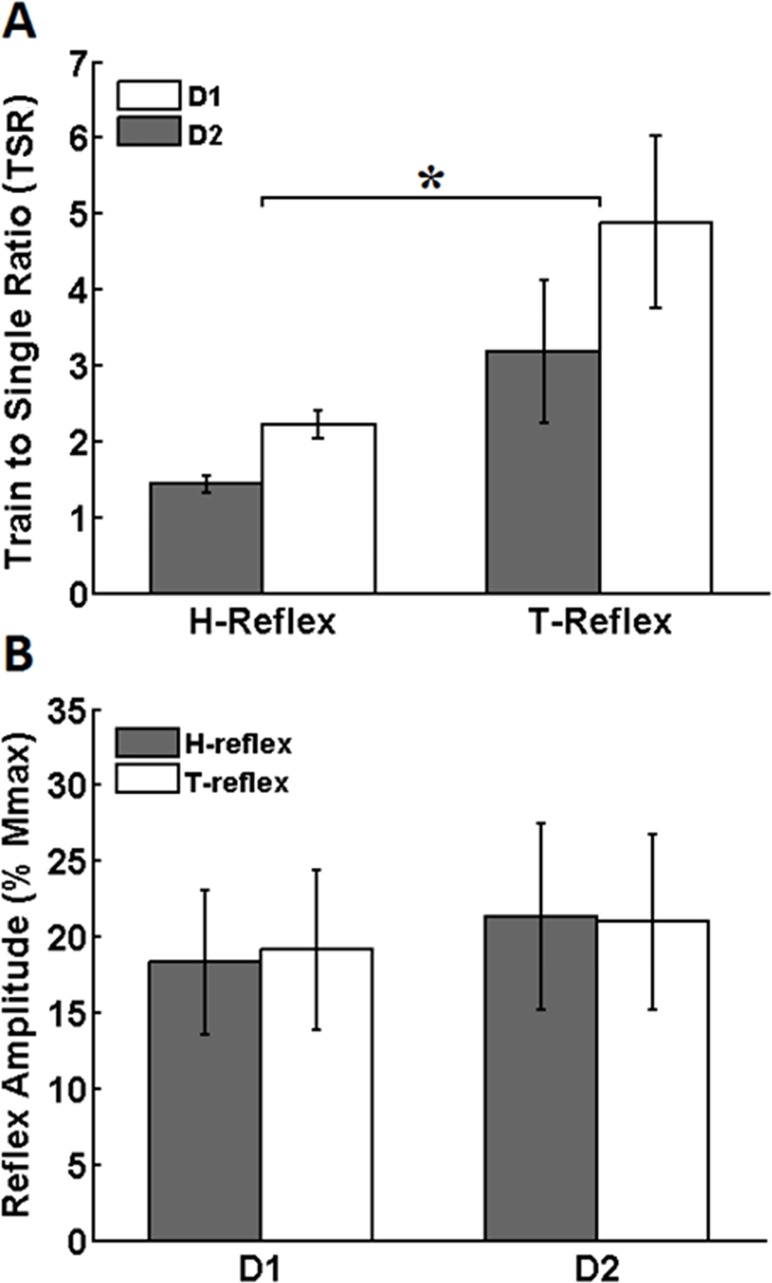
Train to single ratio (TSR) and control responses. A) Ratio between AOI_train and AOI_singl (TSR) for H- and T-reflexes. TSR is significantly higher for T-reflexes (p = 0.022), regardless of the C-T interval used to induce PSI (D1 or D2). This means that, in relation to the inhibition induced by the “single” conditioning stimulus, the amount of inhibition (AOI) under train conditioning is higher for T-reflexes as compared to H-reflexes. Asterisks indicate significant differences between conditions. B) Mean amplitude of control H- and T-reflexes (normalized by Mmax). There were no differences between control reflex amplitudes among different conditions.

The averaged TSR values for both T- and H- reflexes and for both C-T intervals are shown in Fig. 4A. Note the larger TSR values for the T-reflexes as compared with those of the H-reflexes (for both D1 and D2 inhibitions). The two-way ANOVA test performed on TSR revealed a significant main effect for the factor “type of reflex” (F_(1,12)_ = 6.885, p = 0.022). No main effect was found for the factor “type of inhibition (D1 *vs* D2)” (F_(1,12)_ = 2.119, p = 0.171) and no interaction was found between these two factors (F_(1,12)_ = 0.305, p = 0.591). These results indicate that, with respect to the inhibition induced by a single conditioning stimulus, the inhibitory effect of the “train” conditioning was stronger on T-reflexes in comparison to H-reflexes, irrespective of the type of C-T interval.

It is worth mentioning that the 2 subjects that were excluded from the TSR analyses (see Methods) showed qualitatively similar responses to those reported here for group data, i.e. a relatively stronger effect of the “train” condition for T-reflexes as compared to the H-reflexes. Therefore, the exclusion of 2 subjects from the TSR analyses did not bias the related results and interpretations.

Fig. 4B shows the averaged amplitudes of the control reflexes (expressed in %Mmax). Note that control T- and H- reflex were consistently maintained at ~20% Mmax during the experiments. The two-way ANOVA test performed on the amplitude of the control (unconditioned) H- and T- reflexes revealed no main effects for the factors “type of reflex” (F_(1,16)_ = 0.205, p = 0.657) and “type of inhibition” (F_(1,16)_ = 0.897, p = 0.358). Additionally, no significant interaction was found between these two factors (F_(1,16)_ = 1.008, p = 0.330). These results indicate that the control reflexes did not change considerably between trials and conditions.

## Discussion

The present study further explored the sensitivity of H and T reflexes to two important inhibitory mechanisms of reflex modulation (D1 and D2). Surprisingly, the T-reflex was more responsive to trains of conditioning stimuli when compared to single pulses than was the H-reflex. This result was observed for both D1 and D2 inhibitions. Some putative spinal cord mechanisms behind these effects are discussed in what follows.

### Possible mechanisms for the enhanced PSI effect

The results obtained from both H- and T-reflexes show that the conditioning train of pulses induced an increased inhibition when compared with a single conditioning pulse. This was expected for H-reflexes with C-T interval of ~20 ms (D1 inhibition) as previously reported [21]. However, for the other conditions we examined, the results are new and hence add to the present knowledge in the literature. A brief general suggestion on possible mechanisms behind the facilitation of PSI in healthy and post-stroke subjects was presented by Roche et al. [21]. The authors suggested an imbalance between excitatory and inhibitory synaptic actions along the PSI pathway. On the other hand, Lamy et al. [29] suggested that the increased PSI levels they obtained for trains of pulses in the conditioning pathway could be due to a mechanism similar to post-tetanic potentiation which would occur at rates such as 1 Hz. But the interpretation of our results (see below) will lead to a quite different suggestion because post-tetanic potentiation has not been reported at such low rates of stimulation.

It is tempting to suggest that a cumulative facilitation is responsible for the enhanced transmission along the inhibitory presynaptic pathway in response to a train of conditioning stimuli. Nevertheless, such a cumulative effect due to a spatio-temporal summation in interneurons interposed in this pathway is highly unlikely, as the conditioning stimuli were delivered at a very low rate (1 Hz). For the same reason, post-tetanic potentiation should be discarded as a possible synaptic mechanism [11, 30].

Another possibility is a long-loop effect from different types of conditioning stimuli that lead to increased cortical excitability [31, 32]. The descending influence from both corticospinal and reticulospinal fibers can suppress the activity of first or last-order interneurons within the PSI pathway, i.e., they decrease the PSI level, instead of increasing it as currently observed [33–35].

Post-activation depression (or homosynaptic depression—HD), which is the only known synaptic phenomenon with long duration (even longer than 10 s) [36], can be a possible mechanism responsible for the observed results. This depression is presumably caused by a decrease in synaptic transmission due to depletion or decreased probability of release of neurotransmitter [37–40].

Experiments conducted in both humans and cats have shown that HD level is not the same for the whole set of Ia terminals within the spinal cord [29, 41]. It is suggested that the phenomenon of HD is not ascribed solely to the pre-synaptic neuron (Ia afferent) but possibly to an interaction between both pre and post-synaptic cell [29, 42]. Lamy et al. [29] reported that the synapse between Ia terminals and inhibitory Ia interneuron (that mediates reciprocal inhibition at lumbar level) is susceptible to HD. Hence, one may speculate that the depression in synaptic contacts with inhibitory interneurons interposed in the inhibitory pathway can, eventually, lead to a facilitation of PSI.

**Fig 5:**
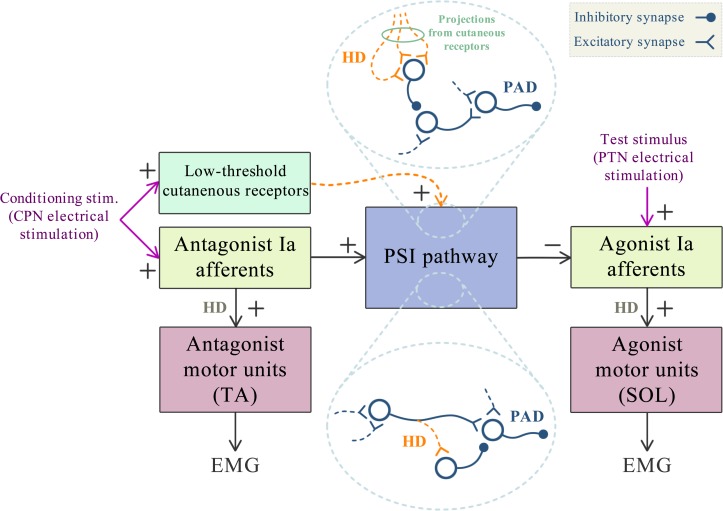
Scheme showing a putative mechanism for the enhancement of PSI due to a low frequency (1 Hz) train of conditioning stimuli. The hypothesis is the occurrence of HD in the transmission to inhibitory interneurons within the neural circuit that generates PSI (bottom circled inset). Another possibility is the occurrence of HD at synapses from low threshold cutaneous afferents that project to inhibitory interneurons whose activation lead to a decrease in the PSI level (top circled inset). The final result from the action of both processes is an increased excitability of the last order (PAD) interneuron.

The architecture of the PSI neuronal network has not yet been fully disclosed, but it is hypothesized that interneurons that mediate primary afferent depolarization (PAD) from muscle afferents are located within the intermediate zone of the lumbosacral region [43]. Inhibitory interneurons in this region that receive direct or indirect contacts from peripheral and/or descending inputs [44], arguably operate as modulators of the PSI pathway. Fig. 5 summarizes the proposed mechanism: the HD in synapses on inhibitory interneurons interposed within the PSI pathway (detailed in the circled inset at the bottom of the figure) might play an important role in the enhanced effect of PSI induced by repetitive conditioning stimuli at 1 Hz. Furthermore, low threshold cutaneous afferents eventually recruited by the conditioning stimulus activate inhibitory interneurons whose action down regulates the PSI level (detailed in the inset at the upper part of Fig. 5) [34, 35]. These afferents could also be subject to HD thereby increasing the PSI level (Fig. 5).

Apart from the neuronal architecture and/or the afferent fibers being stimulated, it seems reasonable to consider a mechanism based on HD to explain the observed enhanced PSI level. An additional experimental protocol that directly assessed the contribution of conditioning stimulation frequency has been devised in the present study. The preliminary results obtained from 3 subjects clearly suggested an increment in the PSI level when a double pulse was applied at 1.5 Hz as compared to 0.5 Hz (Figure A in S1 File). As HD is dependent on the stimulus frequency [45], these preliminary results reinforce the hypothesis of HD being an important mechanism mediating the observed increases in PSI level.

The inhibitory effects observed here were qualitatively similar for both intervals between conditioning and test stimuli. One possibility is that they are part of the same inhibitory pathway, and a ‘facilitatory’ period between D1 and D2 could be ascribed to contamination from a cutaneous pathway [33]. However, the similar inhibitory effects can still be conveyed by different subsets of interneurons. Specific experiments in cats, for example, are necessary to unravel possible differences between both inhibitory processes in terms of neural circuitry.

### Enhanced PSI effect on T-reflexes

Results from post stimulus time histogram in humans indicated that the later part of the excitatory post-synaptic potential (EPSP) is not affected by PSI when a Ia afferent fires at high frequency (due to muscle stretch), which might be the reason for the observed lower sensitivity of T-reflexes to PSI as compared to H-reflexes in C-T paradigms [13]. Enríquez-Denton et al. [14] argued that the accumulation of Ca^2+^ in SO Ia synaptic buttons due to high frequency firing contributes to more neurotransmitter release and to lessen the effect of PSI (compared to a case when there is only one Ia spike reaching the terminal, i.e. during the generation of an H-reflex). However, as our data showed that PSI was enhanced, we propose that conditioning stimulation with trains leads to disinhibition of the pathway mediating PAD, decreasing to a larger extent (as compared to the “single” condition) the influx of Ca^2+^ thereby reducing neurotransmitter release [14].

Supposedly, the ensued higher PAD excitability would be the result of firing in pre-PAD excitatory interneurons released from the control of the inhibitory interneurons (Fig. 5). The increased activity of the PAD interneurons would thoroughly reduce the amplitude of the EPSP. As a final result, the conditioning train would be effective in enhancing the effect of PSI with the consequent reduction in the amplitude of the T-reflex.

One interesting result presented here is that, although the “single” effect was weak for the T-reflex, the effect of the “train” condition was substantial. This was reflected by the train-to-single ratio (TSR) that was more prominent for T-reflexes as compared to H-reflexes. For instance, the values shown in Fig. 4A indicate that the D1 effect for T-reflexes was nearly 5 times higher under the “train” conditioning than under the “single” conditioning, while for H-reflexes the “train” conditioning caused a relative inhibition ~2 times higher as compared to “single”. This result might be explained by the number of afferents activated by the tendon percussion as compared to electrical nerve stimulation.

H and T-reflexes have a different proportion of Ia afferents participating in the generation of responses with similar sizes. This means that a lower number of Ia afferents will produce T-reflex amplitudes similar to those of an H-reflex generated by a comparatively larger number of Ias [11, 13, 46]. As the test stimulus intensity was carefully controlled and kept constant along the experiment for both H and T-reflexes, a proportionally stronger inhibitory effect could, in principle, be expected on a T-reflex as compared to H-reflex. This was found only when the inhibition was caused by a train of stimuli, putatively due to complex interactions between the inhibitory mechanisms and those related to the burst of Ia discharges associated with the tendon tap (as previously described).

Nevertheless, it is quite reasonable to assume that the mechanical and electrical test stimuli activate somewhat different sets of Ia afferents (with diverse firing rates), representing an additional factor that accounts for the presently observed differential effect on the PSI level.

We aknowledge that the techniques used here are not refined enough to provide a definitive explanation. Further experiments (preferably using animal preparations and/or computational modeling) are necessary to disclose the mechanisms responsible for the observed results.

### Functional significance

The T-reflex seems to better represent the functioning of the stretch reflex pathway than the H-reflex, since the former also depends on the muscle-spindle responsiveness and it is associated with a few asynchronous Ia discharges in each axon instead of a single synchronized discharge of all the activated Ia axons [3]. During the performance of a smooth motor task, the excitability of the stretch reflex pathway is constantly being modulated by peripheral and descending signals [6, 47] so the action of the Ia afferents will change smoothly from one level to another. Nonetheless, for brisk movements or when a limb encounters an unexpected obstacle there may occur an Ia afferent firing burst equivalent to that of a tendon tap. The functional significance of the interaction between mechanically-evoked repetitive discharge of Ia afferents and PSI has been discussed [13]. It was proposed that PSI is modulated according to Ia afferent discharge, so that when a sudden muscle stretch takes place PSI is partially suppressed, allowing an appropriate reflex reaction [13].

The present results corroborate and extend previous studies, which reported that low frequency activation of afferents from the antagonist muscle is able to increase the level of PSI onto Ia terminals of the agonist [21, 29]. We propose that these volleys from antagonist Ia afferents (as induced by a low-frequency train of stimuli) might not occur during a motor behavior, but the HD in different synapses within the polysynaptic inhibitory pathway (or any other mechanism triggered by the low frequency stimulation that changes synaptic transmission across the interneurons within this path) will be changing dynamically and hence modulating the PSI level.

Therefore, a general scenario emerges as the reduction in PSI during the high frequency afferent discharge of the agonist (homonymous) muscle (see Morita et al. [13]) might be counteracted by a low frequency volley from the antagonist muscle. It is conceivable that this reciprocal mechanism may act on the stabilization of the ankle joint during a perturbation by promoting a balanced inhibitory action, which depends on the state of the antagonist muscle. One example of such condition is the alleged increased PSI of SO Ia terminals when the subject walks on a narrow base, which requires co-contraction of both ankle flexors and extensors [48]. Alternatively, given the slow time course of the train effect, this mechanism could also play a role during either upright postural maintenance or gait, by ensuring coordinated muscle activation within the limb.

The strength of this modulation depends on the firing behavior of the afferents from the antagonist muscle. It has been stated that the presence of two independent mechanisms with different time courses (namely HD and PSI) provides flexibility for the control of Ia afferent transmission [49]. We presently suggest that this concurrent control, which assists in the modulation of the stretch reflex excitability, is not restricted to the homonymous synapse but may also act at a premotoneuronal level.

One must bear in mind, however, that most of the mechanisms behind the observed effects cannot be directly assessed with the techniques currently employed in humans and either functional or physiological interpretations must be done with reservations. Even with these clear limitations, the present investigation opens the possibility of studying the effects of PSI on the stretch reflex pathway and possible interactions with the fusimotor system [8, 49, 50]. Further, it brings new elements both to re-evaluate a previously reported lack of sensitivity of the T-reflex to PSI and to discuss the functional role of the stretch reflex pathway during a given motor task.

## Supporting Information

S1 File Report of extra-experiments that explored the frequency-dependent effect of the conditioning stimulation on H- and T-reflexes. (PDF)
