# Supplementary material for: Enhanced D1 and D2 Inhibitions Induced by Low-Frequency Trains of Conditioning Stimuli: Differential Effects on H- and T-Reflexes and Possible Mechanisms
Source: PLoS One. 2015 Mar 25;10(3):e0121496. doi: 10.1371/journal.pone.0121496 (PMC4373906; doi:10.1371/journal.pone.0121496)
Supplement: S1 File — (PDF) [file pone.0121496.s001.pdf]

Supporting Information

Enhanced D1 and D2 inhibitions induced by low-frequency trains of conditioning stimuli: differential effects on H- and T-reflexes and possible mechanisms.

Rinaldo André Mezzarane; Fernando Henrique Magalhães; Vitor Martins Chaud;

Leonardo Abdala Elias; André Fabio Kohn

This supplementary material describes an additional set of experiments that was conducted in order to explore whether the enhanced effect of PSI induced by trains of conditioning stimuli is sensitive to the frequency of the conditioning stimulation. The basis for these additional experiments was that a frequency-dependent inhibitory effect (induced by double pulses of conditioning stimulation) would indicate the existence of HD along the PSI pathway. These additional experiments were performed in three subjects. All of them had participated in the main experiments and showed a strong facilitation of PSI elicited by the train of conditioning stimulation.

The protocol employed in these additional experiments mimicked the one described in the paper, except that the trials consisted of single, double pulses at 0.5 Hz and double pulses at 1.5 Hz conditioning stimuli randomly applied to the CPN. Only D2 inhibition was explored in these experiments (i.e. C-T interval of 100 ms). Descriptive statistics were used to compare the amplitude of H- and T-reflexes conditioned by a single stimulus with the amplitude of reflexes conditioned by double stimulation at 0.5 and 1.5 Hz. The amplitude of the reflexes was expressed as a percentage of the mean amplitude observed in the single condition, as shown in Figure A.

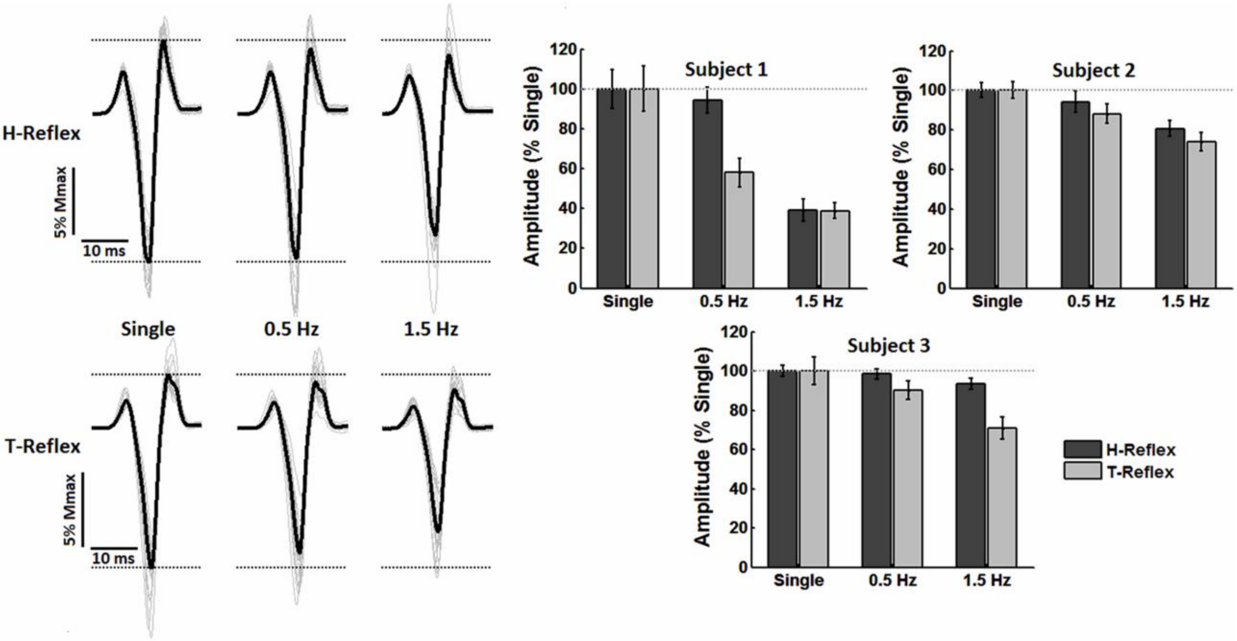

Figure A. Frequency-dependent effect of the conditioning stimulation. Left panel: H- and T-reflexes conditioned by either single or double pulses (delivered at 0.5Hz or 1.5Hz) obtained from one representative subject. Reflex responses conditioned by double pulse at the highest frequency (1.5Hz) showed the greatest PSI. Right panel: Averaged amplitude values (normalized by the mean amplitude observed in the single condition) from all three subjects, showing the frequency-dependence of reflex responses to conditioning. A relatively higher PSI level is noticed for T-reflexes as compared to H-reflexes. Vertical traces indicate SEM.

The results show that, in comparison to the single conditioning, reflex responses were decreased when double pulses of conditioning stimuli were applied. Conditioning stimulation at 1.5 Hz caused a larger reduction of the reflex responses in comparison to the 0.5 Hz conditioning for both types of reflexes. Moreover, in comparison to H-reflexes, T-reflexes showed a larger reduction when double pulses (0.5 and 1.5 Hz) were applied, reinforcing the findings of the experiments reported in the paper (i.e. larger TSRs for T-reflexes as compared to H-reflexes).
